# High prevalence of adverse drug reactions among oral contraceptive pill users in Western India: a prospective observational study

**DOI:** 10.3389/fphar.2026.1925241

**Published:** 2026-10-28

**Authors:** Aishwarya Nitin Dhakne, Bhagyashri Rajopadhye, Vasundhara A. Londhe, Priti Dhande

**Affiliations:** Department of Pharmacology, Bharati Vidyapeeth Deemed to be University Medical College, Pune, Maharashtra, India

**Keywords:** adverse drug reactions (ADRs), combined oral contraceptives (COCs), drug safety, India, oral contraceptive pills (OCPs), pharmacovigilance, polycystic ovary syndrome (PCOS), prospective observational study

## Abstract

Oral contraceptive (OC) pills are among the most widely prescribed hormonal preparations globally, yet prospective adverse drug reaction (ADR) surveillance data from the Indian subcontinent remain sparse. This prospective observational study aimed to estimate the prevalence, spectrum, and temporal pattern of ADRs among OC pill users at a tertiary care gynecology outpatient department and affiliated private clinics in Pune, Western India. A total of 201 women aged 18–60 years prescribed OC pills for contraceptive or non-contraceptive indications were enrolled; 130 were new initiators and 71 were existing users. Participants were followed for 3 months, with ADRs documented at three pre-specified time points anchored to the date of OC pill initiation. Of 201 participants, 179 (89.05%; 95% Wilson confidence interval: 83.98%–92.66%) experienced at least one ADR, with a mean of 1.87 ± 1.06 ADRs per participant. Nausea was the most frequently reported ADR (62.69%), followed by weight gain (25.87%), breast pain (24.88%), vomiting (22.39%), and headache (19.90%). A notable 87.06% of participants used OC pills for non-contraceptive therapeutic purposes, with polycystic ovary syndrome as the predominant indication (36.82%). Among new initiators, a distinct biphasic temporal pattern was observed: early-onset gastrointestinal ADRs—particularly nausea and vomiting—resolved completely by the third month, while weight gain emerged as a persistent late-onset effect. On multivariable analysis, reported weight gain was independently associated with baseline overweight/obese status rather than with polycystic ovary syndrome or OC pill formulation. Serious ADRs were rare, with no thromboembolic events recorded. These findings demonstrate a high ADR burden among OC pill users in an Indian clinical setting, predominantly driven by transient gastrointestinal symptoms, and highlight the importance of anticipatory counseling on the biphasic ADR trajectory, and prospective pharmacovigilance in guiding individualized prescribing.

## 1 Introduction

Oral contraceptive pills (OCPs) constitute one of the most widely prescribed pharmacological interventions in reproductive medicine, with an estimated 151 million women utilizing them globally at any given time (United Nations, 2019). In India, data from the National Family Health Survey-5 (NFHS-5, 2019–21) indicate a modern contraceptive prevalence of approximately 56.5% among currently married women, with oral pills representing the predominant spacing method across urban populations (International Institute for Population Sciences, 2021). The escalating uptake of OCPs within the Indian subcontinent, driven by expanding national family planning initiatives and an increasing breadth of gynecological indications, necessitates rigorous characterization of their adverse drug reaction (ADR) profile within the local clinical context.

OCPs principally comprise two pharmacological categories: combined oral contraceptives (COCs), containing both a synthetic estrogen—most commonly ethinyl estradiol (EE)—and a progestin; and progestin-only pills (POPs), which contain a progestogen alone. The primary contraceptive mechanism of COCs involves negative feedback suppression of the hypothalamic-pituitary-ovarian (HPO) axis; EE inhibits follicle-stimulating hormone (FSH) secretion, restricting follicular development, while the progestin component attenuates the mid-cycle luteinizing hormone (LH) surge, thereby preventing ovulation (Cooper and Patel, 2025). Additional mechanisms include progestin-mediated thickening of cervical mucus, reduced sperm penetrability, and induction of endometrial atrophy (Achache and Revel, 2006). The pharmacokinetics of OCPs are mechanistically central to their ADR profile. EE undergoes extensive first-pass hepatic metabolism via the CYP3A4 enzyme system, resulting in a reported oral bioavailability of approximately 38%–48% (Cooper and Patel, 2025; De Leo et al., 2013). Hepatic induction of coagulation factor synthesis by EE, alongside acquired activated protein C resistance, constitutes the principal pathophysiological mechanism underlying the venous thromboembolism (VTE) risk classically associated with COC use (ASRM, 2016).

The ADR profile of OCPs spans a clinically significant spectrum. Thromboembolic ADRs represent the most serious category; meta-analyses confirm an approximately 1.6-fold elevated relative risk of myocardial infarction and ischemic stroke among COC users compared with non-users (Roach et al., 2015). A nationwide Danish prospective cohort study involving over two million women demonstrated a doubling of the adjusted rate ratio for both ischemic stroke and myocardial infarction (Yonis et al., 2025). Metabolic consequences include dose-dependent dyslipidemia and clinically relevant elevation in blood pressure (Krauss and Burkman, 1992; Laddha et al., 2025). Psychiatric ADRs have attracted increasing attention; a UK Biobank analysis of 264,557 women demonstrated an elevated depression hazard ratio (HR 1.71) during the first 2 years of OC use (Johansson et al., 2023). Current OCP use is associated with a 20%–24% relative increase in breast cancer risk (Hadizadeh et al., 2025).

Despite the breadth of pharmacovigilance data available from Western and European cohorts, prospective ADR surveillance data from the Indian subcontinent remain sparse. Available Indian data document nausea, menstrual irregularities, and weight gain as predominant reported complaints (Samanta and Mondal, 2025; Ray and Sovani, 2006). Systematic, clinic-based, prospective quantification of ADR incidence across the full clinical spectrum has not been adequately addressed in this population (Samanta and Maiti, 2022). This deficit is compounded by consistently high OCP discontinuation rates attributable to adverse effects; Rosenberg and Waugh (1998) demonstrated that approximately 46% of discontinuations were side-effect driven (Rosenberg et al., 1995).

This prospective observational study was therefore designed to systematically estimate the prevalence and characterize the spectrum of ADRs in female subjects using OCP therapy at a tertiary care center and private outpatient facilities in Pune, India, over a three-month observation period, thereby contributing clinically contextualized pharmacovigilance evidence to inform prescribing practices and patient counseling within the Indian healthcare setting.

## 2 Materials and methods

### 2.1 Study design and setting

This was a prospective observational study conducted at the Obstetrics and Gynecology outpatient department (OPD) of a tertiary care teaching hospital (Bharati Vidyapeeth Deemed to be University Medical College, Pune) and affiliated private gynecology clinics in Pune, Maharashtra, India. The study was conducted over a 12-month period from March 2024 to February 2025.

### 2.2 Participants

Women aged 18–60 years who were prescribed oral contraceptive (OC) pills for any indication—contraceptive or non-contraceptive—and presented at the study sites during the enrollment period were eligible for inclusion. The cohort comprised two groups: new initiators, who were freshly prescribed OC pills at enrollment and followed prospectively, and existing users, who were already taking OC pills at enrollment and for whom ADR data across the follow-up interval were obtained by structured history. For both groups, all follow-up time points were anchored to the date of OC pill initiation rather than the date of enrollment, ensuring a consistent time origin. Participants were required to be able to recall and report ADR events experienced during OC pill use. Women with pre-existing chronic conditions that could confound ADR attribution—including hypertension, diabetes mellitus, chronic kidney disease, and chronic liver disease—were excluded.

### 2.3 Sample size estimation

The sample size was calculated using the formula for estimation of a single proportion: n = Z^2^ × P(1−P)/d^2^, where Z = 1.96, P = 0.566 (reference ADR prevalence of 56.6% from a prior study) (Alsayed et al., 2022), and d = 0.07. The minimum required sample size was 193 participants. A total of 201 participants were enrolled.

### 2.4 Data collection and follow-up

Data were collected using a structured, pre-designed proforma capturing sociodemographic characteristics, anthropometric and vital parameters (weight, height, body mass index [BMI], blood pressure, pulse rate, hemoglobin), gynecologic history, and OC pill use details. Anthropometric and vital parameters were recorded at baseline only; serial follow-up measurements were not performed. Each participant was followed for 3 months from the date of OC pill initiation. Follow-up assessments were scheduled at a baseline assessment and three pre-specified follow-up time points relative to OC pill initiation: Day 7 (first follow-up), 1 month (second follow-up), and 3 months (third follow-up). Follow-up was conducted via direct OPD visits or telephonically; telephonic contacts consisted of structured verbal inquiry without physical examination. At each contact, participants were queried regarding new or ongoing adverse effects and compliance. All reported ADRs were documented as present or absent. Reported weight gain was defined as a participant-reported, self-perceived increase of at least 5% over baseline weight; objective serial weight measurements were not obtained. Severity grading and objective quantification of other ADRs (e.g., headache frequency, volume of vaginal bleeding) were not performed.

### 2.5 Outcome assessment

The primary outcome was the prevalence of ADRs over the three-month follow-up period. Incident hypertension was defined as new-onset systolic blood pressure ≥140 mmHg and/or diastolic blood pressure ≥90 mmHg recorded at a clinic follow-up visit in a participant documented as normotensive at baseline, confirmed on the same visit. The secondary outcome was the proportion of participants using OC pills for contraceptive versus non-contraceptive indications. No formal causality assessment tool (e.g., the Naranjo algorithm or the WHO–Uppsala Monitoring Centre system) was applied; ADRs were attributed on the basis of temporal association with OC pill use, known pharmacological profiles, and clinical judgment. Risk factors for ADR occurrence were evaluated using univariable and multivariable logistic regression, with odds ratios (OR) and 95% confidence intervals (CI) reported.

### 2.6 Statistical analysis

Data were compiled in Microsoft Excel and analyzed using IBM SPSS Statistics Version 26 (IBM Corp., Armonk, NY, United States) and Python 3 (statsmodels). Categorical variables were expressed as frequencies and percentages; continuous variables as mean ± standard deviation, median, and interquartile range. The 95% confidence interval for overall ADR prevalence was calculated using the Wilson score method. Chi-square or Fisher’s exact tests were used for categorical associations. Univariable logistic regression was performed to identify candidate risk factors, followed by multivariable logistic regression entering OC pill brand, age group, BMI category, total duration of use, socioeconomic class, indication group, and user type simultaneously. Because several categories exhibited complete separation (100% ADR occurrence), Firth’s penalized-likelihood logistic regression was used for the multivariable models. A separate multivariable model examined determinants of reported weight gain, adjusting for polycystic ovary syndrome status, BMI, OC pill brand, age, and user type; a p-value < 0.05 was considered statistically significant.

### 2.7 Ethical approval

The study was conducted in accordance with the Declaration of Helsinki. Ethical approval was obtained from the Institutional Ethics Committee of Bharati Vidyapeeth Deemed to be University Medical College, Pune (Approval No. [PLACEHOLDER]). The study was registered with the Clinical Trials Registry of India (CTRI Registration No. [PLACEHOLDER]). Informed verbal consent was obtained from all participants prior to enrollment; a waiver of written consent was approved by the Institutional Ethics Committee in view of the observational, non-interventional nature of the study. Confidentiality was maintained throughout; no personal identifiers were used in analysis or reporting.

## 3 Results

### 3.1 Participant characteristics

A total of 201 women were enrolled and completed the three-month follow-up (Figure 1). Of these, 130 (64.7%) were new initiators followed prospectively from OC pill initiation, and 71 (35.3%) were existing users for whom follow-up data were obtained by structured history relative to their initiation date. The mean age was 30.34 ± 7.17 years (median 30; IQR 25–34; range 18–57). Most had university or postgraduate education (71.14%). The moderate socioeconomic class predominated (60.70%). The mean BMI was 23.71 ± 3.95 kg/m^2^, with 68.16% having normal BMI and 27.37% overweight or obese. Mean hemoglobin was 11.48 ± 1.22 g/dL. Baseline characteristics are summarized in Table 1; anthropometric and vital parameters in Supplementary Table S1.

**FIGURE 1:**
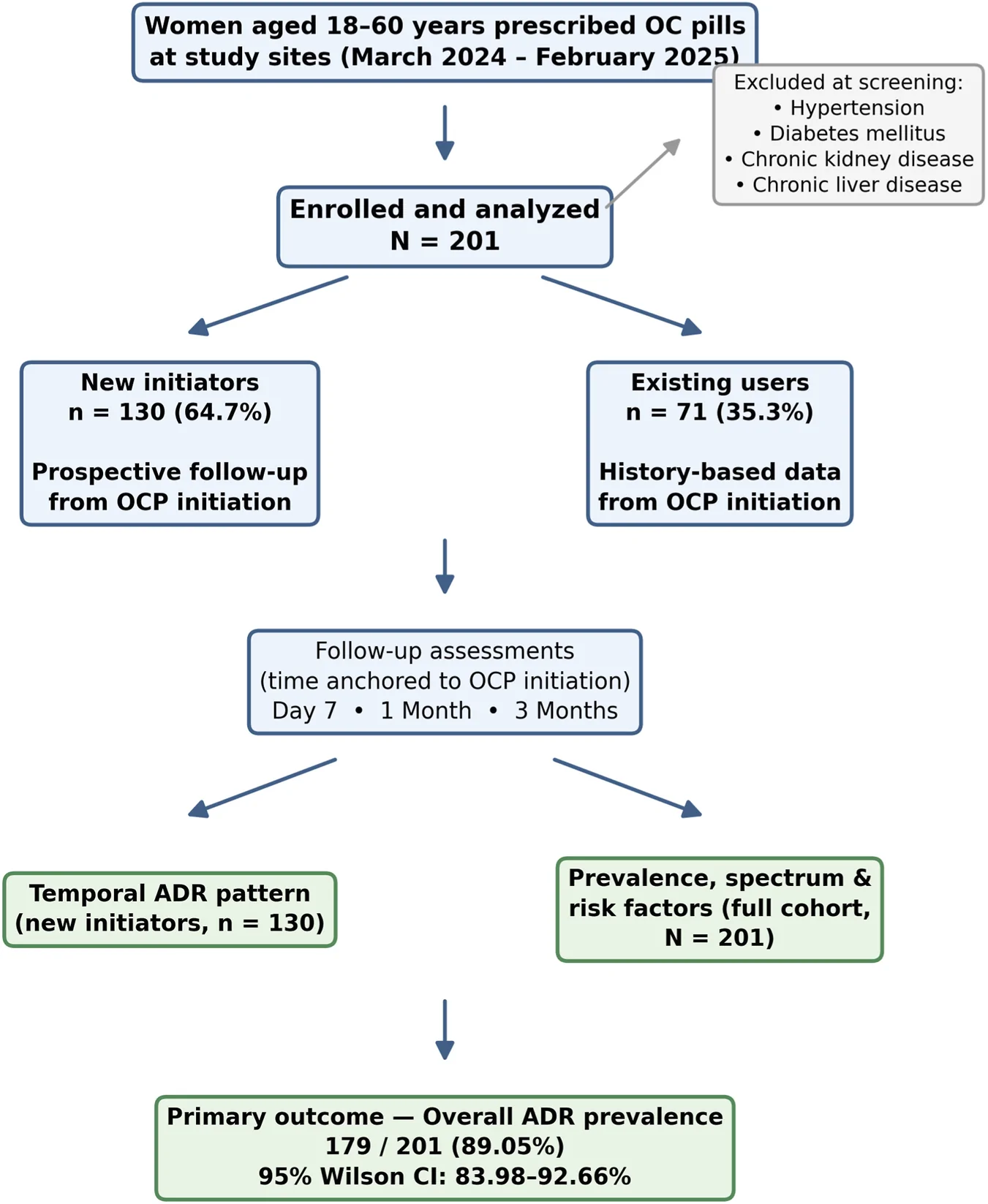
Temporal pattern of adverse drug reactions across three follow-up visits among new initiators (n = 130). Grouped bars represent the prevalence (%) of selected adverse drug reactions and the proportion of participants reporting no adverse drug reactions at each follow-up time point (Day 7, 1 month, and 3 months), anchored to oral contraceptive pill initiation. A distinct biphasic pattern is evident: early-onset gastrointestinal symptoms (nausea, vomiting) declined progressively and resolved by the third follow-up, while weight gain emerged as a persistent late-onset effect. By the third follow-up, 66.9% of new initiators were free of adverse drug reactions.

**TABLE 1:** Baseline characteristics of study participants (N = 201).

Parameter	n/value	%
Age (years)—mean ± SD	30.34 ± 7.17	​
Median (IQR)	30 (25–34)	​
18–25 years	52	25.87
26–30 years	57	28.36
31–35 years	51	25.37
36–40 years	24	11.94
41–60 years	17	8.46
Education—university/postgraduate	143	71.14
Middle/high school	55	27.36
Read and write	3	1.49
Occupation—not working	116	57.71
Working	85	42.29
Socioeconomic class—low	55	27.36
Moderate	122	60.70
High	24	11.94
Regular physical activity—yes	114	56.72
No	87	43.28
BMI (kg/m^2^)—mean ± SD	23.71 ± 3.95	​
Underweight (<18.5)	7	3.48
Normal (18.5–24.9)	137	68.16
Overweight (25–29.9)	37	18.41
Obese (≥30)	18	8.96
Hemoglobin (g/dL)—mean ± SD	11.48 ± 1.22	​

### 3.2 Oral contraceptive pill use patterns

Nearly all participants (99.50%) were prescribed combined estrogen-progesterone formulations. Mala N, a second-generation levonorgestrel-containing preparation, was the most commonly prescribed brand (48.76%), followed by Femilon (24.38%), Ginette 35 (8.96%), Novelon (6.97%), Krimson 35 (4.98%), and Doris (4.98%). Ginette 35 and Krimson 35 are cyproterone acetate–ethinylestradiol preparations indicated primarily for androgen-dependent conditions rather than as first-line contraceptives (Supplementary Table S2). A striking 87.06% of participants were using OC pills for non-contraceptive therapeutic indications, while only 12.94% used them for family planning. Polycystic ovary syndrome (PCOS) was the single most common indication (36.82%), followed by abnormal uterine bleeding (AUB; 14.93%) and primary infertility (11.44%). OC pill use patterns are detailed in Table 2.

**TABLE 2:** Oral contraceptive pill use patterns and indications (N = 201).

Parameter	n	%
OC pill brand—Mala N	98	48.76
Femilon	49	24.38
Ginette 35	18	8.96
Novelon	14	6.97
Krimson 35	10	4.98
Doris	10	4.98
Yamini	1	0.50
Evalon	1	0.50
OC pill type—estrogen–progesterone	200	99.50
Estrogen only	1	0.50
Duration of use—1–2 months	24	11.94
3–4 months	92	45.77
5–6 months	69	34.33
7–8 months	14	6.97
>8 months	2	1.00
Purpose—non-contraceptive	175	87.06
Contraceptive	26	12.94
Indication—PCOS	74	36.82
AUB	30	14.93
Primary infertility	23	11.44
Ovarian Cyst	13	6.47
Menorrhagia	10	4.98
Adenomyosis	8	3.98

### 3.3 Prevalence and spectrum of adverse drug reactions

Of 201 participants, 179 (89.05%; 95% Wilson CI: 83.98%–92.66%) experienced at least one ADR. ADR prevalence was higher among existing users (69/71, 97.18%) than new initiators (110/130, 84.62%); this crude difference was not independently significant after multivariable adjustment (Section 3.6). The mean number of ADRs per participant was 1.87 ± 1.06. Nausea was the most frequently reported ADR (62.69%), followed by weight gain (25.87%), breast pain/tenderness (24.88%), vomiting (22.39%), and headache (19.90%). Less frequent ADRs included depression/anxiety (4.48%), acne (2.99%), diarrhea (2.49%), hypertension (0.50%), and fibroadenoma (0.50%). The complete distribution is presented in Table 3 and Figure 2.

**TABLE 3:** Prevalence and frequency of individual adverse drug reactions (N = 201).

Adverse drug reaction	n	%
Overall ADR prevalence (95% wilson CI: 83.98%–92.66%)	179	89.05
Nausea	126	62.69
Weight gain	52	25.87
Breast pain/tenderness	50	24.88
Vomiting	45	22.39
Headache	40	19.90
Back pain	27	13.43
Abnormal vaginal bleeding	14	6.97
Depression/anxiety	9	4.48
Acne	6	2.99
Diarrhea	5	2.49
Hypertension	1	0.50
Fibroadenoma	1	0.50
ADRs per subject—mean ± SD	1.87 ± 1.06	​
0 ADRs	22	10.95
1 ADR	45	22.39
2 ADRs	85	42.29
3 ADRs	38	18.91
4 ADRs	8	3.98
5 ADRs	3	1.49

**FIGURE 2:**
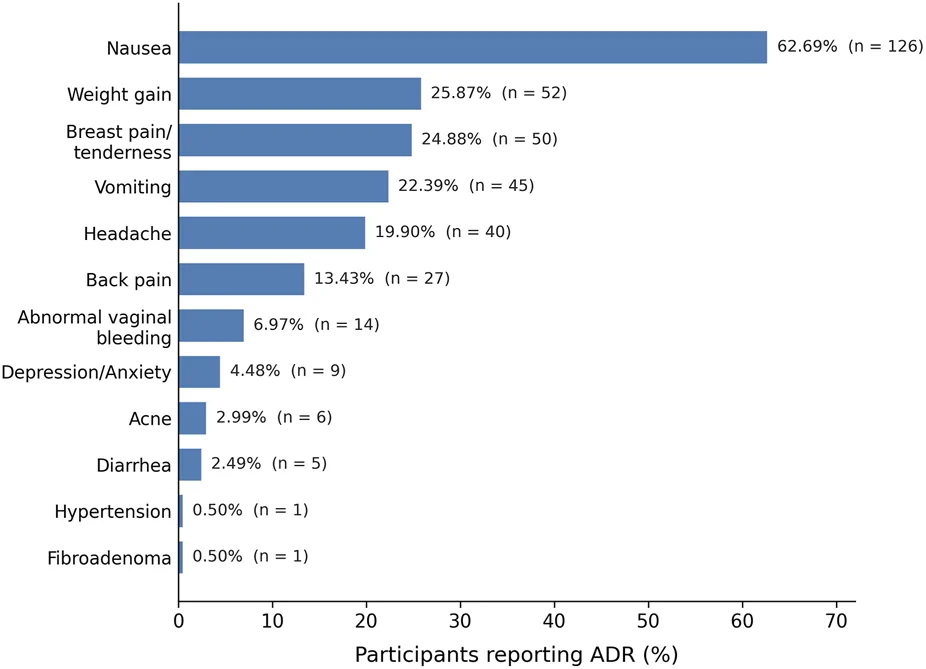
Frequency distribution of individual adverse drug reactions among oral contraceptive pill users (N = 201). Horizontal bars represent the prevalence (%) of each adverse drug reaction reported during the three-month follow-up period; absolute participant counts (n) are indicated adjacent to each bar. Nausea was the most frequently reported adverse drug reaction (62.69%), with gastrointestinal symptoms collectively affecting nearly two-thirds of the cohort.

### 3.4 Temporal pattern of ADRs across follow-up visits

To ensure a valid time origin, the temporal analysis was restricted to the 130 new initiators, for whom follow-up was prospective from OC pill initiation. A distinct biphasic temporal pattern emerged (Table 4; Figure 3). At the first follow-up (Day 7), nausea was dominant (60.8%), with breast tenderness (17.7%), vomiting (20.8%), and headache (11.5%). By the second follow-up (1 month), nausea declined to 15.4%, vomiting resolved completely, and weight gain emerged prominently (23.8%). By the third follow-up (3 months), the majority (66.9%) reported no ADRs; nausea, vomiting, and diarrhea had resolved, while weight gain persisted in 23.1%. Early-onset gastrointestinal ADRs were self-limiting, while weight gain emerged as a late-onset persistent effect.

**TABLE 4:** Temporal pattern of adverse drug reactions across three follow-up visits among new initiators (n = 130).

ADR	Follow-up 1 (Day 7) n (%)	Follow-up 2 (1 Month) n (%)	Follow-up 3 (3 Months) n (%)
Nausea	79 (60.8)	20 (15.4)	0 (0.0)
Vomiting	27 (20.8)	0 (0.0)	0 (0.0)
Breast tenderness	23 (17.7)	8 (6.2)	1 (0.8)
Weight gain	0 (0.0)	31 (23.8)	30 (23.1)
Headache	15 (11.5)	11 (8.5)	6 (4.6)
Back pain	2 (1.5)	5 (3.8)	3 (2.3)
Depression/anxiety	3 (2.3)	2 (1.5)	1 (0.8)
Acne	2 (1.5)	1 (0.8)	1 (0.8)
Abnormal bleeding	8 (6.2)	3 (2.3)	1 (0.8)
Diarrhea	0 (0.0)	1 (0.8)	0 (0.0)
No ADRs	30 (23.1)	52 (40.0)	87 (66.9)

The temporal analysis is restricted to the 130 new initiators, for whom follow-up was prospective from the date of oral contraceptive pill initiation.

**FIGURE 3:**
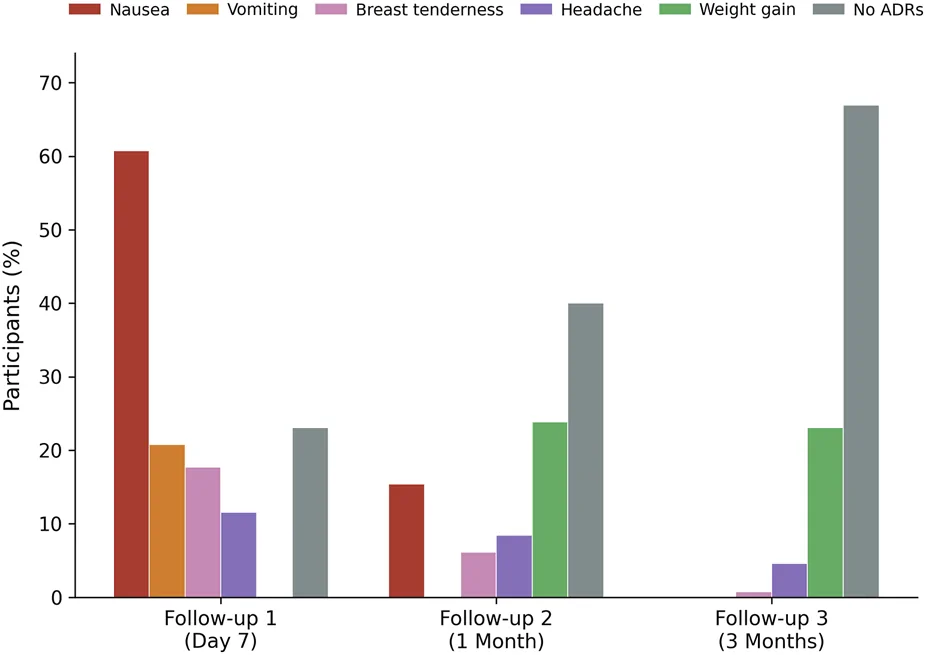
Participant enrollment and analytical flow. A total of 201 women were enrolled and completed the three-month follow-up. Participants comprised 130 new initiators (64.7%), followed prospectively from oral contraceptive (OC) pill initiation, and 71 existing users (35.3%), for whom data were obtained by structured history relative to initiation. The temporal ADR analysis was restricted to new initiators, whereas prevalence, spectrum, and risk-factor analyses used the full cohort. Overall ADR prevalence was 179/201 (89.05%; 95% Wilson confidence interval 83.98%–92.66%).

### 3.5 Association of ADRs with clinical and demographic variables

The association between individual ADRs and OC pill brand was analyzed across the six major brands prescribed (Table 5). On univariable testing, five ADRs showed statistically significant brand-dependent variation: breast pain (p = 0.0018), headache (p = 0.0006), back pain (p = 0.0142), nausea (p = 0.0426), and vomiting (p = 0.0101). Breast pain showed a significant association with age group, being highest in the 31–35 years group (37.3%; p = 0.0425). The ADR profile was broadly similar between contraceptive and non-contraceptive user groups.

**TABLE 5:** Association of individual adverse drug reactions with oral contraceptive pill brand (N = 201).

ADR	Mala N (98)	Femilon (49)	Ginette 35 (18)	Novelon (14)	Krimson 35 (10)	Doris (10)	p
Breast pain	11 (11.2)	18 (36.7)	6 (33.3)	4 (28.6)	4 (40.0)	5 (50.0)	0.0018*
Headache	13 (13.3)	7 (14.3)	8 (44.4)	3 (21.4)	1 (10.0)	6 (60.0)	**0.000** 6*
Back pain	5 (5.1)	8 (16.3)	6 (33.3)	3 (21.4)	1 (10.0)	2 (20.0)	0.0142*
Nausea	58 (59.2)	34 (69.4)	8 (44.4)	8 (57.1)	10 (100)	8 (80.0)	0.0426*
Vomiting	25 (25.5)	6 (12.2)	1 (5.6)	3 (21.4)	5 (50.0)	5 (50.0)	0.0101*
Weight gain	24 (24.5)	12 (24.5)	9 (50.0)	2 (14.3)	2 (20.0)	3 (30.0)	0.2353
Depression	3 (3.1)	2 (4.1)	3 (16.7)	0 (0.0)	1 (10.0)	0 (0.0)	0.1314
Abn. bleeding	4 (4.1)	5 (10.2)	2 (11.1)	2 (14.3)	0 (0.0)	1 (10.0)	0.4755
Fibroadenoma	0 (0.0)	0 (0.0)	0 (0.0)	0 (0.0)	0 (0.0)	1 (10.0)	0.0019*
Diarrhea	2 (2.0)	1 (2.0)	0 (0.0)	1 (7.1)	1 (10.0)	0 (0.0)	0.4975
Acne	2 (2.0)	2 (4.1)	2 (11.1)	0 (0.0)	0 (0.0)	0 (0.0)	0.3471
Hypertension	0 (0.0)	1 (2.0)	0 (0.0)	0 (0.0)	0 (0.0)	0 (0.0)	0.6882

Values are n (%). *p < 0.05 (chi-square or Fisher’s exact test). Brand-wise associations should be interpreted in the context of confounding by indication (see Supplementary Table S2).

Bold values indicate statistically significant associations (p < 0.05).

### 3.6 Risk factors for ADR occurrence

On univariable analysis, several OC pill brands, longer total duration of use, and socioeconomic class were associated with ADR occurrence; however, Femilon, Ginette 35, the pooled minor-brand group, and the 5–6 months duration category each showed 100% ADR occurrence, producing complete separation and unstable maximum-likelihood estimates (Table 6). Firth-penalized multivariable logistic regression was therefore used (Table 7). After simultaneous adjustment, only Femilon retained a statistically significant association with any ADR (adjusted OR = 21.07; 95% CI: 1.54–287.44; p = 0.022), with a wide confidence interval reflecting the small subgroup size; the Ginette 35 (adjusted OR = 5.90; p = 0.30) and pooled minor-brand (adjusted OR = 7.96; p = 0.11) associations were no longer significant. User type was not an independent predictor (adjusted OR = 0.86; p = 0.90), indicating that the crude prevalence difference between existing and new users was explained by other covariates. Duration of use represents total cumulative OC pill exposure, including use prior to enrollment. Given the separation and small per-category counts, brand and duration estimates should be interpreted with caution, and no formulation can be reliably ranked for tolerability from these data.

**TABLE 6:** Univariable logistic regression for any adverse drug reaction (N = 201).

Variable	Category	N	ADR n (%)	OR	95% CI	p
Pill brand	Mala N	98	76 (77.6)	Reference	—	—
​	Femilon	49	49 (100)	29.12	1.73–491.05	0.0001*
​	Ginette 35	18	18 (100)	10.88	0.63–187.77	0.0222*
​	Others	36	36 (100)	21.47	1.27–363.86	0.0010*
Duration (total)	1–2 months	24	17 (70.8)	Reference	—	—
​	3–4 months	92	79 (85.9)	2.50	0.87–7.21	0.1251
​	5–6 months	69	69 (100)	59.57	3.24–1093.94	<0.0001*
​	≥7 months	16	14 (87.5)	2.88	0.51–16.15	0.2717
Socioeconomic	High	24	24 (100)	Reference	—	—
​	Moderate	122	110 (90.2)	0.18	0.01–3.15	0.2168
​	Low	55	45 (81.8)	0.09	0.00–1.57	0.0274*
Age group	18–25	52	44 (84.6)	Reference	—	—
​	26–35	108	102 (94.4)	3.09	1.01–9.44	0.0686
​	36–60	41	33 (80.5)	0.75	0.25–2.21	0.7828

*p < 0.05. Several categories (Femilon, Ginette 35, Others, 5–6 months) showed 100% ADR, occurrence, producing complete separation and unstable maximum-likelihood estimates. See Table 7 for the Firth-penalized multivariable analysis. Duration represents total cumulative OC, pill exposure including pre-enrollment use.

Bold values indicate statistically significant associations (p < 0.05).

**TABLE 7:** Univariable and multivariable (firth-penalized) logistic regression for any adverse drug reaction.

Variable	Category	Univariable OR (95% CI)	Univ. p	Adjusted OR (95% CI)†	Adj. p
Pill brand	Mala N	Reference	​	Reference	​
​	Femilon	29.12 (1.73–491.05)	0.0001	21.07 (1.54–287.44)	0.0223*
​	Ginette 35	10.88 (0.63–187.77)	0.0222	5.90 (0.21–165.24)	0.2964
​	Others	21.47 (1.27–363.86)	0.0010	7.96 (0.61–103.94)	0.1135
Age group	18–25	Reference	​	Reference	​
​	26–35	3.09 (1.01–9.44)	0.0686	1.02 (0.31–3.31)	0.9793
​	36–60	0.75 (0.25–2.21)	0.7828	0.53 (0.17–1.67)	0.2772
BMI	Normal/UW	Reference	​	Reference	​
​	OW/Obese	1.02 (0.38–2.76)	1.0000	0.93 (0.34–2.54)	0.8901
Duration	1–2 months	Reference	​	Reference	​
​	3–4 months	2.50 (0.87–7.21)	0.1251	1.95 (0.62–6.10)	0.2517
​	5–6 months	59.57 (3.24–1093.94)	<0.0001	9.74 (0.36–262.05)	0.1754
​	≥7 months	2.88 (0.51–16.15)	0.2717	1.63 (0.07–38.00)	0.7626
Socioeconomic	High	Reference	​	Reference	​
​	Moderate	0.18 (0.01–3.15)	0.2168	2.60 (0.13–51.42)	0.5295
​	Low	0.09 (0.00–1.57)	0.0274	3.96 (0.17–91.91)	0.3915
Purpose	Contraceptive	Reference	​	Reference	​
​	Non-contracep	0.65 (0.14–2.94)	0.7454	1.12 (0.22–5.62)	0.8881
User type	New initiator	Reference	​	Reference	​
​	Existing user	—	—	0.86 (0.09–8.41)	0.8982

† Adjusted odds ratios from Firth penalized logistic regression (all variables entered simultaneously; N = 199 complete cases). Firth’s method was used because standard maximum-likelihood estimation failed due to complete separation. Wide confidence intervals reflect small subgroup sizes and residual near-separation; adjusted brand and duration estimates are hypothesis-generating.

Bold values indicate statistically significant associations (p < 0.05).

Because weight gain is a recognized feature of PCOS, a separate multivariable model examined its determinants (Table 8). The crude association between PCOS and reported weight gain (OR = 1.56; 95% CI: 0.82–2.98; p = 0.18) was fully attenuated after adjustment (adjusted OR = 1.12; 95% CI: 0.45–2.78; p = 0.81). Baseline overweight/obese status was the only significant independent predictor of reported weight gain (adjusted OR = 5.98; 95% CI: 2.92–12.24; p < 0.0001), indicating that weight gain reported during OC pill use was attributable to baseline adiposity rather than to PCOS or OC pill formulation.

**TABLE 8:** Determinants of reported weight gain: Firth-penalized multivariable analysis (N = 199).

Variable	Crude OR (95% CI)	Adjusted OR (95% CI)	Adj. p
PCOS (vs. non-PCOS)	1.56 (0.82–2.98)	1.12 (0.45–2.78)	0.8119
BMI overweight/obese (vs. Normal/UW)	—	5.98 (2.92–12.24)	<0.0001*
Brand: Femilon (vs. Mala N)	—	1.38 (0.52–3.66)	0.5137
Brand: Ginette 35 (vs. Mala N)	—	3.77 (0.85–16.64)	0.0798
Brand: Others (vs. Mala N)	—	0.95 (0.30–3.01)	0.9289
Age 26–35 (vs. 18–25)	—	0.56 (0.21–1.48)	0.2432
Age 36–60 (vs. 18–25)	—	0.64 (0.21–1.95)	0.4365
Existing user (vs. new)	—	0.71 (0.30–1.68)	0.4355

The crude association between PCOS, and reported weight gain (OR, 1.56) is fully attenuated after adjustment for BMI (adjusted OR, 1.12, p = 0.81). Baseline overweight/obese status is the only significant independent predictor, indicating that weight gain reported during OC, pill use is attributable to baseline adiposity rather than PCOS, or OC, pill formulation.

Bold values indicate statistically significant associations (p < 0.05).

## 4 Discussion

### 4.1 Participant profile and prescribing context

The present cohort of 201 women (mean age 30.34 ± 7.17 years) is demographically representative of the reproductive-age population attending tertiary care gynecology services in urban India. The predominantly normal-weight profile (mean BMI 23.71 kg/m^2^) is clinically significant, as BMI is an independent risk factor for VTE among COC users and may partly explain the absence of thromboembolic events during the study period (Chatsatourian et al., 2025; Nguyen et al., 2024). The most distinctive feature of this study is the overwhelmingly non-contraceptive indication for OC pill use (87.06%), with PCOS the single most common indication (36.82%). This contrasts sharply with Western cohorts where contraception predominates and positions the present findings as particularly relevant to the evolving therapeutic landscape of OCPs in low- and middle-income settings(Salem et al., 2024).

### 4.2 ADR prevalence and spectrum

A total of 179 of 201 participants (89.05%) experienced at least one ADR, exceeding the prevalence reported in most comparable studies: Karpowicz et al. (2024) documented 79.2%, Rothschild et al. (2022) 71%, and Alsayed et al. (2022) 62.9%. This higher prevalence likely reflects the prospective, structured follow-up and the predominantly non-contraceptive population, many of whom were symptomatic at baseline (Alhagbaker et al., 2022). The higher crude ADR prevalence among existing users (97.18%) than new initiators (84.62%) is compatible with cumulative exposure and with a survivor effect, whereby women who tolerate OC pills poorly discontinue and are less likely to be captured as ongoing users; this difference was not independent of other covariates on adjustment (Gupta et al., 2024). Nausea was the most frequently reported ADR (62.69%), with gastrointestinal symptoms collectively affecting nearly two-thirds of participants. Weight gain (25.87%) was consistently identified across comparable studies (Goresh et al., 2025; Ibrahim et al., 2019). Depression and mood-related changes were reported by only 4.48%—substantially lower than international estimates—likely reflecting cultural reluctance to disclose mood symptoms and the absence of standardized psychiatric screening, and therefore probably an underestimate (Haase et al., 2025;Johansson et al., 2023).

### 4.3 Temporal pattern of ADRs

Restricting the temporal analysis to new initiators, for whom follow-up was prospective from initiation, the study demonstrated a distinct biphasic temporal pattern. Early-onset gastrointestinal and neurological effects predominated at the first follow-up and resolved substantially by 3 months, consistent with the acute pharmacological impact of exogenous steroids and subsequent neuroendocrine adaptation, aligning with Loder et al. (2005) and Kaunitz et al. (2022). In contrast, weight gain emerged as a prominent late-onset ADR, persisting through the third follow-up (Ramadan et al., 2025; Tozetto et al., 2022). This biphasic pattern has direct clinical implications: anticipatory counseling about transient early-onset effects may improve adherence, while vigilance for emerging late-onset metabolic effects should be maintained.

### 4.4 Determinants and risk factors

On univariable analysis, OC pill brand appeared strongly associated with ADR occurrence; however, because Femilon, Ginette 35, and the pooled minor-brand group each had 100% ADR occurrence, these estimates were affected by complete separation and were unstable. After Firth-penalized multivariable adjustment, only Femilon retained a significant, though imprecise, association, and no formulation could be reliably ranked for tolerability. These findings must be interpreted in the context of confounding by indication: brand choice in this cohort was closely tied to clinical indication, age, and clinician preference—for example, cyproterone acetate–ethinylestradiol preparations (Ginette 35, Krimson 35) were prescribed predominantly for hyperandrogenic conditions rather than for contraception (Supplementary Table S2). A small, non-randomized observational study of this design cannot disentangle intrinsic drug tolerability from the characteristics of the patients selected for each formulation. Reviewer-directed multivariable analysis of weight gain further illustrated this point: the crude association between PCOS and weight gain was fully attenuated after adjustment for BMI, with baseline overweight/obese status emerging as the sole independent predictor. This indicates that weight gain reported during OC pill use is driven substantially by baseline adiposity rather than by the drug or the underlying diagnosis, and underscores the importance of multivariable adjustment when attributing ADRs in populations with a high prevalence of metabolic comorbidity.

### 4.5 Severity and clinical significance

The overwhelming majority of ADRs observed were mild and self-limiting. Serious ADRs were rare. One case of incident hypertension (0.50%) was recorded in a 46-year-old woman who was normotensive at baseline and developed a blood pressure of 170/100 mmHg at the first follow-up, prompting OC pill discontinuation—consistent with the established understanding that estrogen-containing COCs increase blood pressure through activation of the renin-angiotensin-aldosterone system (dos Santos et al., 2022). No thromboembolic events were recorded, likely reflecting the young mean age, low obesity prevalence, and short follow-up duration; however, such events may require longer cumulative exposure to manifest (Yonis et al., 2025).

### 4.6 Limitations

Several limitations should be considered. First, the single-center design may limit generalizability. Second, the three-month follow-up is insufficient to detect thromboembolic, oncological, and sustained metabolic effects. Third, the cohort combined new initiators and existing users; although all time points were anchored to OC pill initiation, data for existing users were obtained by history and are subject to recall bias, and the temporal analysis was therefore restricted to new initiators. Fourth, ADRs were ascertained by structured self-report without objective corroboration: anthropometric and vital parameters were measured only at baseline, reported weight gain was defined as a self-perceived increase of at least 5% over baseline weight without serial measurement, and the frequency or severity of other ADRs was not quantified. Fifth, no formal causality assessment tool was applied. Sixth, complete separation in several exposure categories necessitated penalized regression and yielded wide confidence intervals, so brand- and duration-specific estimates are hypothesis-generating rather than definitive. Finally, the study did not include a non-user control group, precluding causal attribution of observed ADRs exclusively to OC pill use.

### 4.7 Future directions

Prospective multi-center studies with follow-up of at least 12–24 months, inclusion of a non-user control arm and progestin-only pill users, integration of validated psychiatric screening, and application of standardized causality assessment tools are needed to build a robust Indian pharmacovigilance evidence base. Given that 87.06% of this cohort used OC pills for non-contraceptive indications, a dedicated analysis focused on this population represents a valuable direction for future work.

## Data Availability

The original contributions presented in the study are included in the article/Supplementary Material, further inquiries can be directed to the corresponding author.

## Supplementary material

Supplementary Table S1 Anthropometric and vital parameters (N = 201).

Supplementary Table S2 Pharmacological composition and classification of prescribed oral contraceptive brands.
