# Supplementary material for: High prevalence of adverse drug reactions among oral contraceptive pill users in Western India: a prospective observational study
Source: Front Pharmacol. 2026 Oct 28;17:1925241. doi: 10.3389/fphar.2026.1925241 (PMC13752540; doi:10.3389/fphar.2026.1925241)
Supplement: Supplementary file 1 [file Table1.docx]

**Supplementary Tables**

**Supplementary Table S1.** Anthropometric and Vital Parameters (N = 201).

| **Parameter** | **Mean** | **SD** | **Median** | **IQR** | **Range** |
| --- | --- | --- | --- | --- | --- |
| Weight (kg) | 60.16 | 10.89 | 58 | 53–65 | 37–97 |
| Height (cm) | 159.28 | 6.08 | 160 | 154–165 | 139–172 |
| BMI (kg/m²) | 23.71 | 3.95 | 22.66 | 21.13–25.66 | 14.7–39.4 |
| Systolic BP (mmHg) | 117.61 | 8.70 | 120 | 110–120 | 94–170 |
| Diastolic BP (mmHg) | 77.21 | 6.02 | 80 | 70–80 | 60–100 |
| Pulse (bpm) | 83.27 | 7.82 | 82 | 80–84 | 66–117 |
| Hemoglobin (g/dL) | 11.48 | 1.22 | 11.8 | 11.00–12.22 | 7.4–15.2 |

*Anthropometric and vital parameters were recorded at baseline only; serial follow-up measurements were not performed.*

**Supplementary Table S2.** Pharmacological Composition and Classification of Prescribed Oral Contraceptive Brands.

| **Brand** | **n (%)** | **Estrogen (dose)** | **Progestin (dose)** | **Generation** | **Primary Approved / Common Indication** |
| --- | --- | --- | --- | --- | --- |
| Mala N | 98 (48.8) | Ethinylestradiol (30 µg) | Levonorgestrel (150 µg) | Second | Contraception (Govt of India subsidized) |
| Femilon | 49 (24.4) | Ethinylestradiol (20 µg) | Desogestrel (150 µg) | Third | Contraception |
| Ginette 35 | 18 (9.0) | Ethinylestradiol (35 µg) | Cyproterone acetate (2 mg) | Anti-androgenic‡ | Hyperandrogenism, acne, hirsutism, PCOS |
| Novelon | 14 (7.0) | Ethinylestradiol (30 µg) | Desogestrel (150 µg) | Third | Contraception |
| Krimson 35 | 10 (5.0) | Ethinylestradiol (35 µg) | Cyproterone acetate (2 mg) | Anti-androgenic‡ | Hyperandrogenism, acne, hirsutism, PCOS |
| Doris | 10 (5.0) | Ethinylestradiol (30 µg) | Drospirenone (3 mg) | Fourth | Contraception, PCOS, PMDD |
| Yamini | 1 (0.5) | Ethinylestradiol (30 µg) | Drospirenone (3 mg) | Fourth | Contraception, PCOS |
| Evalon | 1 (0.5) | Estradiol valerate (2 mg) | Estrogen-only (none) | — | Menopausal HRT / estrogen replacement |

*‡ Cyproterone acetate–ethinylestradiol combinations (Ginette 35, Krimson 35) are anti-androgenic preparations licensed primarily for androgen-dependent conditions rather than as first-line contraceptives, although they confer contraceptive effect. All are 21+7 monophasic regimens. Doses reflect standard Indian market formulations and should be verified against dispensed products. PMDD, premenstrual dysphoric disorder; HRT, hormone replacement therapy.*

*ADR, adverse drug reaction; AUB, abnormal uterine bleeding; BMI, body mass index; CI, confidence interval; IQR, interquartile range; OC, oral contraceptive; OR, odds ratio; OW, overweight; PCOS, polycystic ovary syndrome; UW, underweight. Statistically significant p-values (p < 0.05) are shown in bold and marked with an asterisk.*
